# Pathological complete response to neoadjuvant TACHP in HER2-positive/HR-positive inflammatory breast cancer: a case report

**DOI:** 10.3389/fonc.2026.1768425

**Published:** 2026-03-31

**Authors:** Mengjie Guo, Guangjin Liu, Xin Han, Rui Jiao, Jinmao Li

**Affiliations:** 1 Department of Breast and Thyroid Surgery, Xi’an Daxing Hospital l, Xi’an, Shaanxi, China; 2 Department of Pathology, Xi’an Daxing Hospital, Xi’an, Shaanxi, China

**Keywords:** case report, inflammatory breast cancer, neoadjuvant therapy, pathological complete response, TACHP regimen

## Abstract

Inflammatory breast cancer is a highly aggressive and locally advanced form of breast cancer with a poor prognosis. Neoadjuvant chemotherapy is the cornerstone of current treatment, aiming to downstage the disease and enable surgical intervention, which is typically followed by adjuvant radiotherapy. This paper reports the case of a 48-year-old female patient who presented with a large palpable mass and inflammatory skin changes in her left breast. She was diagnosed with left-sided inflammatory breast cancer (cT4dN2aM0, Stage IIIB, HER2-positive/hormone receptor-positive). Considering the established efficacy of anthracycline and taxane agents in breast cancer treatment, as well as the widespread application of the TAC regimen in neoadjuvant therapy, the patient received six cycles of neoadjuvant TAC chemotherapy combined with dual HER2-targeted therapy (HP). This was followed by a modified radical mastectomy of the left breast. The patient tolerated this intensive regimen well, and postoperative pathological evaluation confirmed a pathological complete response (pCR). This case adds evidence that an intensified TACHP regimen may be feasible and effective in high tumor-burden, HER2-positive inflammatory breast cancer. Therefore, for selected high-risk inflammatory breast cancer patients, the TACHP regimen may be considered as a neoadjuvant treatment option under close monitoring.

## Introduction

Breast cancer is the most common malignancy among women worldwide, with its incidence continuing to rise, posing a significant threat to women’s health (1). Inflammatory breast cancer is a highly aggressive subtype. Although it accounts for only 1-4% of all breast cancer cases, it is characterized by rapid progression and a particularly poor prognosis (2, 3). Its typical clinical presentation includes diffuse breast hardening, erythema, edema, peau d’orange appearance, and warmth affecting at least one-third of the breast skin, which may occur with or without a palpable mass (4–6). These signs result from the obstruction of dermal lymphatic vessels by tumor emboli (7, 8), rather than representing true histological inflammation. Effective treatment remains a significant clinical challenge. This paper reports a case of pCR achieved with the TACHP neoadjuvant regimen in a patient with HER2-positive/HR-positive inflammatory breast cancer, presenting a potential new therapeutic approach for this condition.

For HER2-positive/HR-positive inflammatory breast cancer with high tumor burden, optimal neoadjuvant regimens remain uncertain, and there is limited real-world data on intensified TACHP strategies.

## Case report

### Initial presentation and diagnosis

**Figure 1:**
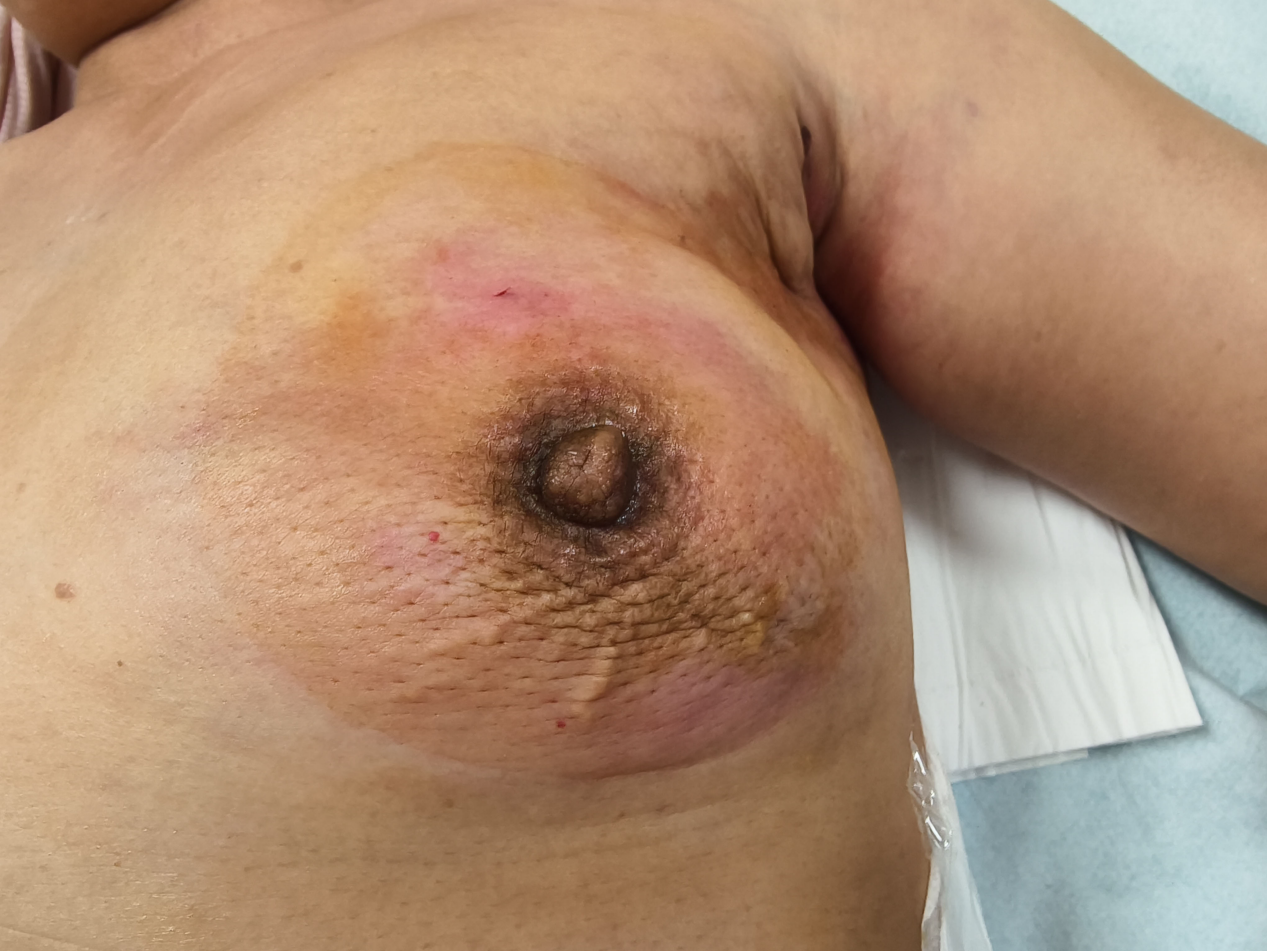
Breast skin erythema and swelling in a patient with inflammatory breast cancer at initial presentation. Written informed consent was obtained from the patient for the publication of the images.

**Figure 2:**
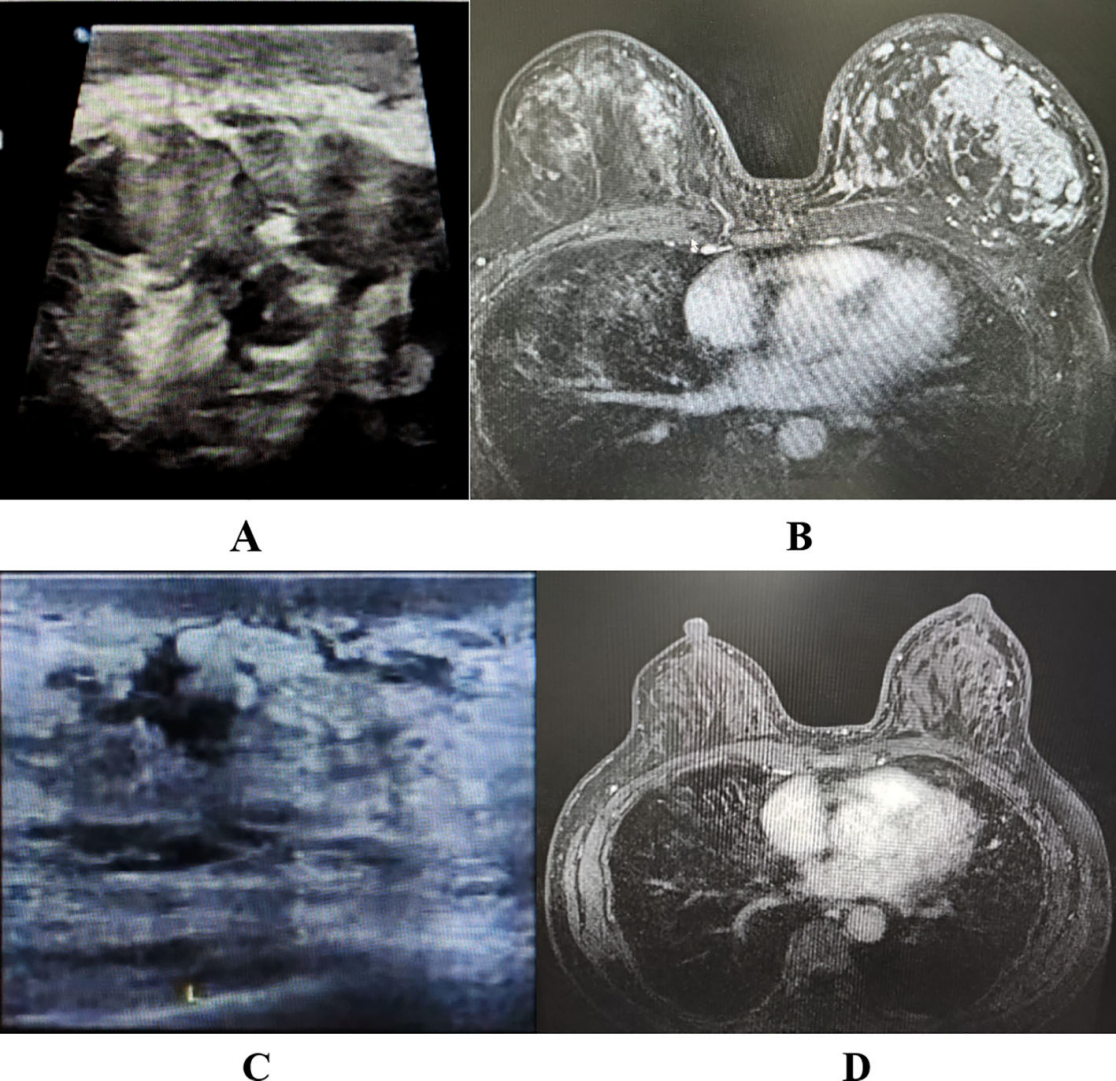
Pre-chemotherapy and post-chemotherapy diagnostic imaging of a patient with inflammatory breast cancer. Pre-treatment ultrasound **(A)** showing edema of the left breast skin and a hypoechoic area within the glandular tissue with indistinct borders and an irregular shape. Pre-treatment MRI **(B)** showing thickening of the left breast skin and multiple heterogeneously enhancing masses with poorly defined, coalescing margins within the glandular tissue, measuring approximately 112 × 45 mm in the largest cross-section. Post-treatment ultrasound **(C)** demonstrating persistent edema of the left breast skin and an irregular, patchy hypoechoic area within the glandular tissue with indistinct borders, showing a reduction in size compared to the pre-treatment scan. Post-treatment MRI **(D)** demonstrating mild skin thickening and multiple nodular signal foci with poorly defined, coalescing margins within the left breast, measuring approximately 35 × 10 mm in the largest cross-section.

A 48-year-old female patient was admitted to our department (Breast and Thyroid Surgery, Xi’an Daxing Hospital) on July 7, 2025, with a chief complaint of “left breast malignancy diagnosed 25 days prior.” She reported no significant family history of cancer or other chronic medical conditions. The diagnosis of invasive carcinoma in the left breast was initially confirmed via core needle biopsy at another hospital in June. She received no further treatment at that time and presented to our department in July. Physical examination revealed breast asymmetry with significant swelling of the left breast. The left nipple was inverted without discharge. The skin over the upper-outer and lower-outer quadrants of the left breast was erythematous, warm to the touch, and associated with an irregular, poorly defined, firm mass approximately 10 × 10 cm in size (**Figure 1**). A notably enlarged, hard, and fixed lymph node was palpable in the left axilla. Breast ultrasound (**Figure 2A**) showed subcutaneous edema in the left breast and several irregular, ill-defined hypoechoic masses, predominantly in the upper-outer and lower-outer quadrants, categorized as BI-RADS 6. Several abnormal, round lymph nodes with thickened cortex and loss of the fatty hilum were observed in the left axilla, the largest measuring approximately 11.8 × 10.5 mm. Breast MRI (**Figure 2B**) demonstrated skin thickening and multiple mass-like lesions within the left breast, which appeared hypointense on T1-weighted and hyperintense on T2-weighted images. These lesions showed marked heterogeneous enhancement with irregular, coalescing borders, the largest spanning approximately 112 × 45 mm in cross-section. Multiple enlarged, intensely enhancing lymph nodes were noted in the left axilla, the largest measuring about 24 × 14 mm, suggestive of metastatic involvement. Core needle biopsies of the left breast mass and a left axillary lymph node were performed in our department. Pathology confirmed invasive breast carcinoma in the breast specimen and metastatic carcinoma in the lymph node. Immunohistochemistry results were as follows: ER (15%), PR (0), HER-2 (2+), and Ki-67 (50%). Fluorescence *in situ* hybridization (FISH) confirmed HER-2 gene amplification. Based on the clinical presentation and pathological findings, the patient was diagnosed with left inflammatory breast cancer (cT4dN2aM0, Stage IIIB, HER2-positive/hormone receptor-positive).

### Neoadjuvant treatment and toxicity

The combination of taxanes and anthracyclines is a well-established treatment for reducing breast cancer recurrence and mortality (9). Furthermore, the TAC regimen is recognized in the Chinese Society of Clinical Oncology (CSCO) Breast Cancer guidelines as a widely applicable neoadjuvant chemotherapy option across various breast cancer subtypes (10). Consequently, for select patients with locally advanced disease (such as the present case), our department initiates TAC chemotherapy promptly upon confirmation of adequate tolerance, even prior to receiving the complete immunohistochemistry report. The regimen consisted of albumin-bound paclitaxel (200 mg), liposomal doxorubicin (50 mg), and cyclophosphamide (0.9 g) on day 1. Upon confirmation of HER2-positive status, dual HER2-targeted therapy was incorporated, with albumin-bound paclitaxel (200 mg), trastuzumab (loading dose 560 mg, then 420 mg), and pertuzumab (loading dose 840 mg, then 420 mg) administered on day 8. This 21-day cycle was repeated for a total of six cycles. The protocol included provisions to switch to TCbHP or THP regimens if the patient experienced significant toxicity or declined to continue the initial therapy. During the six cycles of chemotherapy, supportive care included pegylated recombinant human granulocyte colony-stimulating factor for myelosuppression, glutathione and polyene phosphatidylcholine for liver function management, and ondansetron plus aprepitant for nausea and vomiting. Treatment-related adverse events were assessed according to the Common Terminology Criteria for Adverse Events (CTCAE, version 6.0). The observed hematologic toxicities included grade 2 leukopenia and neutropenia, and grade 1 anemia. Non-hematologic toxicities were limited to grade 1 transaminase elevation and manageable grade 1–2 nausea. No grade 3 or higher non-hematologic toxicities were observed. The lowest recorded laboratory values were as follows: white blood cell count, 2.50 × 10^9/L; neutrophil count, 1.83 × 10^9/L; hemoglobin, 100 g/L. The peak alanine aminotransferase and aspartate aminotransferase levels were 57 U/L and 45 U/L, respectively. Cardiac function remained stable, with a left ventricular ejection fraction of 67% maintained throughout. Pre-operative re-evaluation with breast ultrasound (**Figure 2C**) showed residual subcutaneous edema and several irregular, patchy hypoechoic areas in the left breast, predominantly in the upper-outer and lower-outer quadrants. The largest of these ill-defined lesions measured approximately 15.3 × 8.2 × 12.6 mm and was classified as BI-RADS 6. Several lymph nodes with relatively clear borders and slightly thickened cortex were noted in the left axilla, the largest measuring about 12.0 × 4.3 mm. Breast MRI (**Figure 2D**) indicated mild skin thickening and multiple nodular lesions within the breast parenchyma. These appeared hypointense on T1-weighted and hyperintense on T2-weighted images, demonstrating marked heterogeneous enhancement with irregular, coalescing borders. The largest lesion had a cross-sectional size of approximately 35 × 10 mm. No pathologically enlarged lymph nodes were observed in either axilla. Based on these imaging findings, the clinical assessment was partial response (PR).

### Surgery and pathological findings

**Figure 3:**
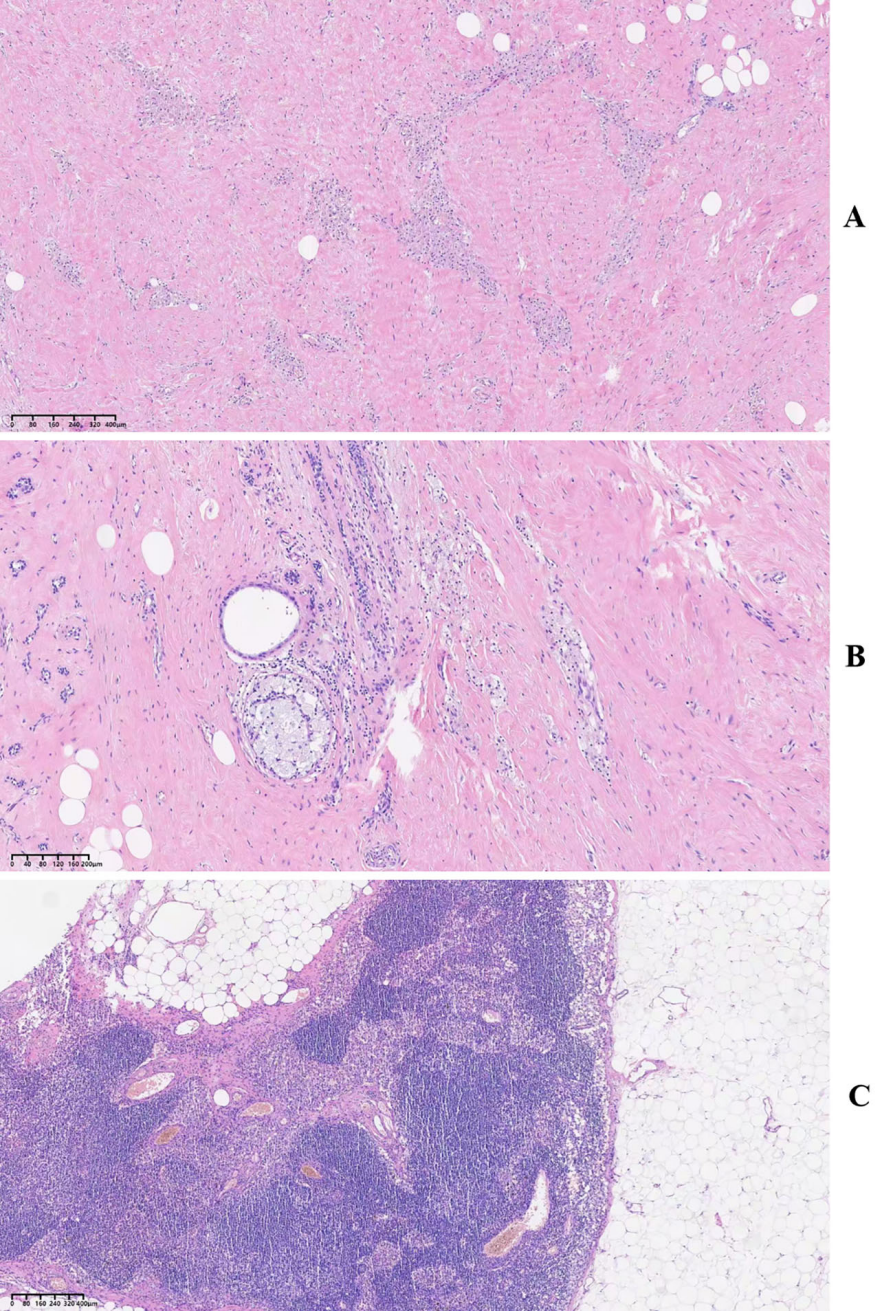
Postoperative pathological findings from a patient with inflammatory breast cancer. **(A, B)** Breast tissue sections showing stromal fibrosis with hyaline degeneration, focally dilated glandular spaces, and aggregation of numerous foam cells, consistent with treatment-related changes. No residual carcinoma is identified. **(C)** Lymph node section showing histiocytic aggregates as a treatment-related effect. No metastatic carcinoma is present.

A left modified radical mastectomy was performed on November 25. Intraoperative frozen section analysis confirmed negative surgical margins. The final pathology report of the resected specimen indicated no evidence of residual invasive carcinoma in the left breast tissue, with only treatment-related changes observed. Examination of the nipple, skin, and basal margins found no tumor involvement. All nine lymph nodes recovered from the left axilla were free of metastatic carcinoma, showing only post-treatment changes. The Residual cancer burden (RCB) index was scored as 0 (**Figure 3**). Based on these pathological findings, the post-treatment stage was ypT0N0cM0, which indicated the achievement of a pCR.

### Post-treatment course/follow-up

Immunohistochemical staining was considered positive for estrogen receptor (ER) or progesterone receptor (PR) if ≥1% of tumor cell nuclei were stained, with 1%–10% staining defined as low expression (11). For this patient with HER2-positive, ER-positive, and PR-negative disease, our department anticipated limited benefit from endocrine therapy and prioritized anti-HER2 treatment. Consequently, the endocrine regimen was limited to oral Toremifene (60mg once daily). After completing the remaining cycles of dual HER2-targeted therapy, treatment was intensified with a sequential tyrosine kinase inhibitor (TKI). The patient also completed a course of 15 radiotherapy sessions between December 26, 2025, and January 14, 2026. The last follow-up was conducted on January 14, 2026.

**Figure 4:**
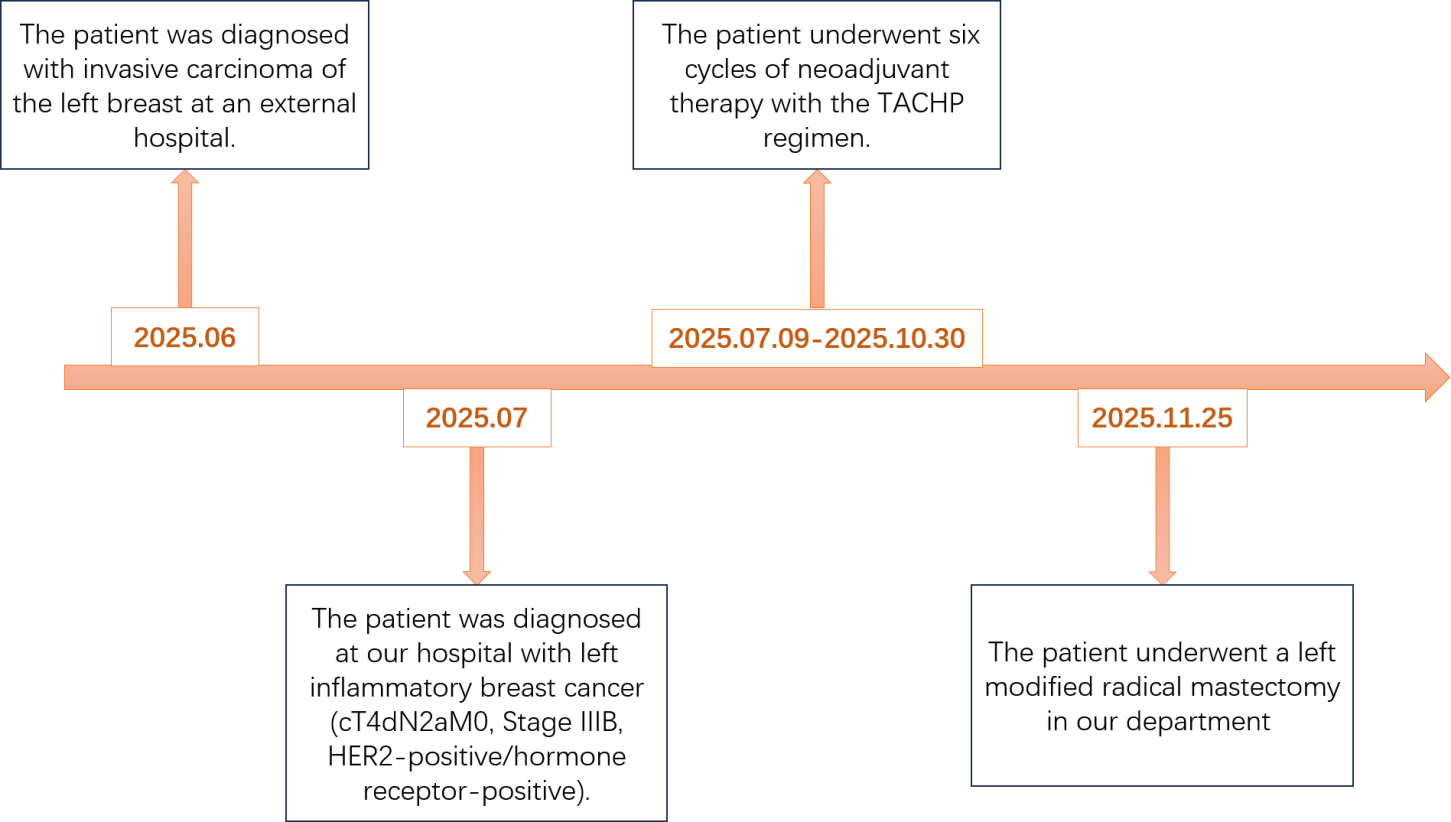
The patient’s detailed treatment course is outlined in the figure. TACHP denotes a combination regimen consisting of nab-paclitaxel (T), liposomal doxorubicin (A), cyclophosphamide (C), trastuzumab (H), and pertuzumab (P).

The patient’s detailed treatment course is outlined in the figure (**Figure 4**). The patient underwent a core needle biopsy at an outside hospital in June 2025 and presented to our center on July 7, 2025. Neoadjuvant therapy was administered from July 9 to October 30, 2025, followed by surgery on November 25, 2025.

## Discussion

This case demonstrates that an intensified TACHP regimen can achieve pCR in a HER2-positive/HR-positive inflammatory breast cancer with extensive tumor burden, with acceptable short-term toxicity. Chemotherapy broadly targets rapidly dividing cells, while targeted therapy inhibits specific molecular pathways in cancer cells. Their combination attacks the tumor through both generalized and precise mechanisms, producing a synergistic effect greater than the sum of its parts across molecular, cellular, and tumor microenvironment levels. The current standard neoadjuvant approach for HER2-positive breast cancer combines chemotherapy with the targeted agents trastuzumab and pertuzumab. The safety and efficacy of the TCbHP regimen have been demonstrated in the KRISTINE (12) and TRAIN-2 (13) trials. Similarly, the therapeutic effectiveness of the THP regimen has been established by studies such as HELEN-006, NeoSphere, and PEONY (14–17). However, due to the inherent cytotoxicity of chemotherapy, treatment de-escalation is an emerging trend. Pooled analysis from three WSG trials showed that neoadjuvant dual HER2-targeted therapy alone achieved a pCR in 31% of selected early-stage patients, with an excellent 5-year overall survival of 97% (18). The PHERGain study further confirmed that chemotherapy can be spared for a subset of these patients (19). It is important to note that these de-escalation strategies have primarily been studied in patients with lower tumor burdens, typically those with stage II to III, non-inflammatory breast cancer. In contrast, this case of inflammatory breast cancer (cT4dN2aM0, stage IIIB) presented with a more advanced stage and higher tumor burden—a population often underrepresented or excluded in the aforementioned trials. For such high-risk patients, opting for conventional chemotherapy or a chemotherapy-free approach would be unsuitable. The TACHP regimen employed in this case represents an individualized intensification of the current standard neoadjuvant therapy for HER2-positive breast cancer. For patients with high tumor burden who are in good general condition and willing to accept greater treatment-related toxicity, the short-term benefits of this approach may substantially outweigh the risks. This strategy aims to maximize tumor cell eradication, rapidly reduce tumor burden, and improve the rate of pCR. Achieving pCR allows subsequent adjuvant endocrine therapy, radiotherapy, and targeted therapy to proceed on a foundation of minimal or no residual disease, which significantly improves prognosis and facilitates subsequent recovery and follow-up.

### Limitations

This study has several limitations. First, as a retrospective analysis of a single case, the evidence level is inherently low, and the findings cannot be generalized. Second, the follow-up duration was short; thus, the long-term efficacy and safety of this intensified regimen require further observation. Third, potential selection bias exists, as the patient was in good general condition, which may have contributed to the tolerability and favorable outcome, and may not represent all patients with inflammatory breast cancer. Finally, treatment decisions were influenced by specific institutional protocols and regional clinical practices, which may limit the broader applicability of the approach.

## Conclusion

In summary, this case report demonstrates that an intensified TACHP neoadjuvant regimen achieved a pCR with manageable toxicity in a patient with high-tumor-burden, HER2-positive inflammatory breast cancer. This outcome suggests that for selected patients with similar high-risk features, such an intensified approach may represent a viable therapeutic option. However, this represents an exceptional case. Currently, sequential administration of anti-HER2 therapy following anthracycline-based chemotherapy remains the standard treatment approach. Concurrent administration of anthracyclines and anti-HER2 agents should only be considered after careful evaluation by specialists and when deemed necessary. Future prospective, randomized controlled trials are needed to definitively assess the efficacy and safety of intensified regimens like TACHP in this challenging patient population.

## Patient perspective

I was admitted due to redness, swelling, and pain in my left breast. Following a diagnosis of breast cancer, I began chemotherapy. During this period, I experienced occasional mild nausea and vomiting without any other significant side effects. After completing chemotherapy, I underwent surgery. The subsequent examination results indicated an excellent treatment outcome.

## Data Availability

The original contributions presented in the study are included in the article/supplementary material. Further inquiries can be directed to the corresponding author.
